# HPV Types Distribution in General Female Population and in Women Diagnosed with Cervical Cancer Across Western Kazakhstan

**DOI:** 10.31557/APJCP.2019.20.4.1089

**Published:** 2019

**Authors:** Saule K Balmagambetova, Andrea Tinelli, Olzhas N Urazayev, Kanshaiym Z Sakieva, Arip K Koyshybaev, Dinara A Zholmukhamedova, Saltanat T Urazayeva

**Affiliations:** 1 *West Kazakhstan Marat Ospanov State Medical University, 68, Maresyev Street, Aktobe, Kazakhstan,*; 2 *Department of Obstetrics and Gynecology, Division of Experimental Endoscopic Surgery, Imaging, Technology and Minimally Invasive Therapy, Vito Fazzi Hospital, Lecce, Italy. *

**Keywords:** Human papillomavirus, types distribution, viral load, cervical cancer, Kazakhstan

## Abstract

**Objective:**

to detect the HPV types distribution both in general female population and in women with first diagnosed cervical cancer, including viral load in both groups.

**Methods:**

Qualitative detection and quantification of HPV was performed by PCR-Real time method based on the Russian equipment and test systems (“DNA-Technology” LLC, Russia). The DNA of low-risk (HPV 6, 11, 44) and possibly / potentially / high carcinogenic risk (HPV 16, 18, 26, 31, 33 , 35, 39, 45, 51, 52, 53, 56, 58, 59, 66, 68, 73, 82), a total of 21 types, were detected.

**Results:**

During the period of 2014-2017 total 1,166 clinically healthy women were tested for HPV, as well as 73 women diagnosed with CaCx. Overall prevalence of HPV in female population was determined as 25.0% (95% CI 22.3;27.7, p = 0.05). Top-5 leading HPV types: 16 (26.4%); 31 (10.1%); 51 (9.4%); 52 (9.0%); 6 (7.9%). Average viral load was 5.5±3.8 (CI 95% 5.1;5.9). In women diagnosed with CaCx ranking was as follows – 16 (54.1%), 31 (11.2%), 18 / 58 (5.1% each), 33 / 45 (4.1% each). Type 16 appeared to be one of the most significant risk factors of the CaCx development (p=0.00007, phi 0.35, Pierson’s *X *^2^ 15.9). Average viral load in patients with CaCx was 6.9±4.0 (95% CI 6.1;7.7). A reliable relationship between the cancer staging and the viral load was found (p = 0.043, n = 73). Domination of type 16 calls for urgent need the transition to HPV primary screening and resumption of immunization program discontinued in 2014. The study is registered in ISRCTN registry, No. ISRCTN71514910 (01.02.2018).

## Introduction

The human papillomaviruses (HPVs) is a large diverse group of viruses of about 190 fully characterized types. In total, 4 groups are currently classified, including group 1, containing types of highly carcinogenic risk (HR-HPV); probably carcinogenic, such as type 68 (group 2a); possibly carcinogenic, such as 26, 53, 66, 67, 70, 73 and 82 types, considered potential carcinogens, with an arguable and still not thoroughly defined role in carcinogenesis (group 2b); not classified (group 3); and, possibly not carcinogenic types (group 4) (Schiffman et al., 2009). Aggregated damage caused by papillomaviruses is estimated about 5% of all cancers of the human body (Mesri et al., 2014). Growing number of proofs on the HPV continuous evolution resulted in clear molecular-biological evidence of the uniquely high carcinogenicity of types currently classified as probably / possibly carcinogenic (Halec et al., 2014; Kim et al., 2014). Leading researchers suggested that for these types of HPV, previous classification should be revised, although there is no need to include them in routine screening tests (Arbin et al., 2014). For activity towards classification revision, foremost the entire body of information on prevalence and leading types causing cervical cancer (CaCx) across all areas of the world is needed. Meanwhile, data on HPV leading types across Kazakhstan still are limited with a few publications (Kairbayev et al., 2013; Bekmukhambetov et al., 2016; Niyazmetova et al., 2016; Aimagambetova et al., 2018). At the same time, situation with the incidence and prevention of CaCx still remains strained in Kazakhstan, despite the existing nationwide screening program. Experts from the Information Center on HPV and Cancer (ICO) stated in 2015 that cervical cancer in Kazakhstan was the second most prevalent among women and ranked first in frequency in the 15-44 age group, with rough incidence rate 32.8 (Bruni et al., 2015). Therefore, making a nationwide map of the HPV prevalence along with identifying the most dangerous types in regard to the cervical cancer is the foreground task for domestic researchers. 

Thus, the present study aims to determine the overall HPV prevalence and types distribution both in general female population and in women with first diagnosed cervical cancer. The study has also provided obtaining findings on HPV viral load, which is well-known predictor of the infection severity, eventually leading to invasive cancer development (Lorincz et al., 2002). 

*Methods*

*Study setting*

The present study constitutes a part of large multipurpose research on the scope of HPV infection which was held in western region of the country during 2014-2017. Full Protocol of the research has been published upon registration in the ISRCTN registry (Bekmukhambetov et al., 2018). The design and Protocol of the study were approved by the University’s Institutional Review Board (09.10.2014). The work was carried out in accordance with the Helsinki Declaration principles. All participants who signed the informed consent form were fully informed on the objectives of this analysis. 

*Population*

When determining the Sample size for general female population, the previous study’s findings mattered - total HPV prevalence 26.04%, p≤0.043, N 1,098 (Bekmukhambetov Y et al., 2016). In total, N was counted as 1,152, of which 417 in Aktobe, 253 in Uralsk, 237 in Atyrau, 245 in Mangystau. 

Data on adult (18+) female population of the republic, along with the age-ajusted incidence of cervical cancer in Kazakhstan were applied to calculate the sample size for having CaCx patients, and the number of 67-80 enrolled was determined. 

Data were collected in medical settings in cities of regional importance, including the nearest vicinities. To reach maximally possible scope of female population and avoid possible bias, all kinds of outpatient clinics were involved: state-sponsored, insurance and private ones. Enrollment of women was held either during their routine visit to the gynecologist, or by ads placed in the clinics lobby, or by the invitation of sentinel specialists. Randomization was not required due to the screening type of study. 

Inclusion criteria for general sample were the following: age 18-60+ years; resident of western Kazakhstan of any ethnicity; no vaccination history.

Exclusion criteria were: non-residents of Kazakhstan; vaccination history. HIV status and pregnancy of I trimester were not Exclusion criteria. 

Inclusion criteria for having CaCx: any age; any stage of the cancer process; histological verification of the diagnosis. Exclusion criteria: non-residents of the Western Kazakhstan; presence of previous medical intervention - radiotherapy, chemotherapy, surgical treatment.

## Materials and Methods

Qualitative detection and quantification of Human papillomavirus was performed by PCR-Real time method based on the Russian test systems and equipment (“DNA-Technology” LLC, Russian Federation). Production of the company “DNA-technology” was certified (ISO 13485: 2012) and registered in the Republic of Kazakhstan (RK-MT-7-No. 013267 dated July 23, 2014). 

*Test systems used in this study*

Kit of the HPV reagents Quantum-21 is designed for identifying, typing and quantifying the DNA of low-risk human papillomavirus (HPV 6, 11, 44) and possibly / potentially / high carcinogenic risk (HPV 16, 18, 26, 31, 33 , 35, 39, 45, 51, 52, 53, 56, 58, 59, 66, 68, 73, 82), total 21 types. To isolate the viral DNA, we used sets of reagents PROBA-NK-PLUS, of the same production. Type of analysis is absolute, that is, a certain number of copies of the virus per sample, measured in genomic equivalents (GE) with a logarithmic calculus. The viral load of up to 103 GE HPV per 100,000 human cells was regarded as “small,” from 10^3^ to 10^5^ GE as “moderate”, and 10^5^ GE and higher as “high” respectively. 

Besides, all assays taken in having CaCx women were subjected to HPV DNA sequencing according to commonly accepted methods (Meneses et al., 2014). DNA isolation for sequencing was performed using a DNA extraction kit Qiagen according to the Manufacturer’s protocol (Spin Protocol). For amplification, the nested PCR method with two pairs of primers was used: the first pair as MY09 / MY11 (Sigma-Aldrich), and the second pair as GP05 + / GP06 + (Sigma-Aldrich). Sequencing of the samples was carried out using the BigDye v3.1 Cycle Sequencing terminator kit.

*Results processing*

All calculations were carried out in the program Statistica.10 (Dell software, USA). For all tests a two side type I error of p=0.05 or less at 95% CI was assumed statistical significant. Non-parametric operational tests were used in this paper due to a priori missing a normal distribution. Analysis to measure the strength of associations between the variables was performed when appropriate (Pearson’s *X *^2^, Cramer’s V). 

## Results

*General sample (common female population)*

During the period of 2014-2017 total 1,166 clinically healthy women were tested for HPV with “Quantum-21” test system. Age/ethnic attributes of the general female population are summarized in Table 1.

**Table 1.** Main Demographic Features of the GeneralSample

By provinces (oblasts)	Detailing	Маngystau	Акtobe	Аtyrau	Uralsk	The whole region
N tested		249	411	242	264	1,166
Age categories	18-29	35.3	43.7	40.5	29.2	37.70%
	30-39	36.1	32.4	29.7	38.3	34.00%
	40-49	20.1	16.2	16.1	19.3	17.80%
	50-63	8.4	7.7	13.6	13.2	10.50%
Average age		34.6±9.4	33.1±9.7	34.6±10.5	35.8±9.9	34.5±9.9 (16.0 – 63.0)
						Мe 33.0 (27.0-41.0
						by 25/75 quartile)
Ethnicity	Representatives of Turkic-speaking people	84.3	87.8	88	80.7	85.30%
	Representatives of the Slavic diasporas, Germans	12	11.6	11.6	19.3	13.60%
	Azerbaijanians, Dagestanians, other Caucasians	3.6	0.6	0.4	0	1.10%

**Figure 1:**
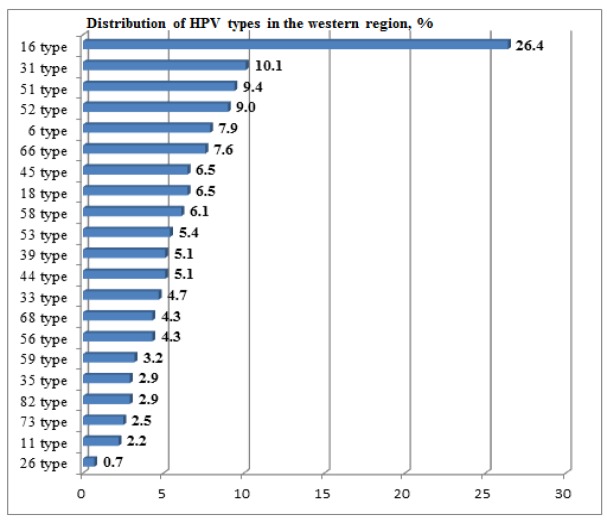
Distribution of Identified HPV Types Throughout the Region

**Figure 2:**
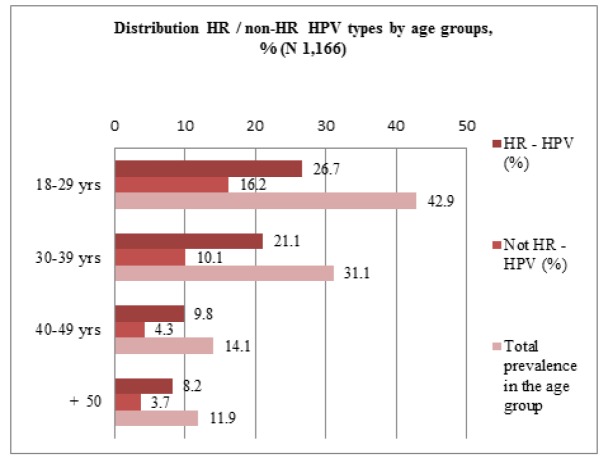
Distribution of HR and non-HR Types by Age Categories

**Figure 3:**
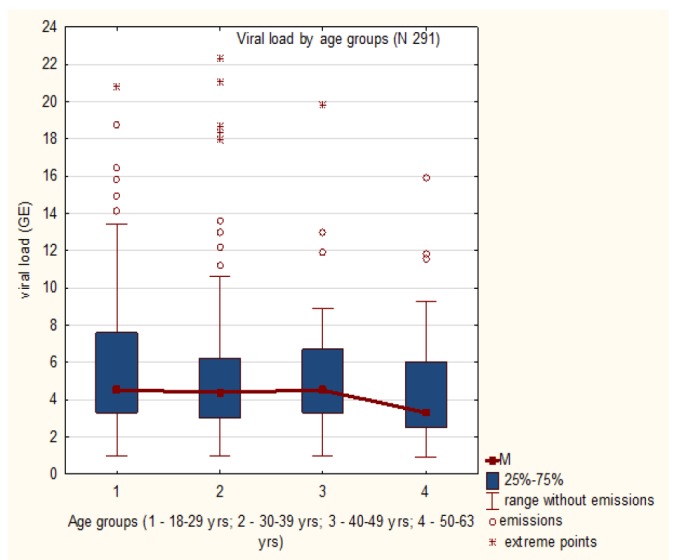
HPV Viral Load by Age Groups

**Figure 4:**
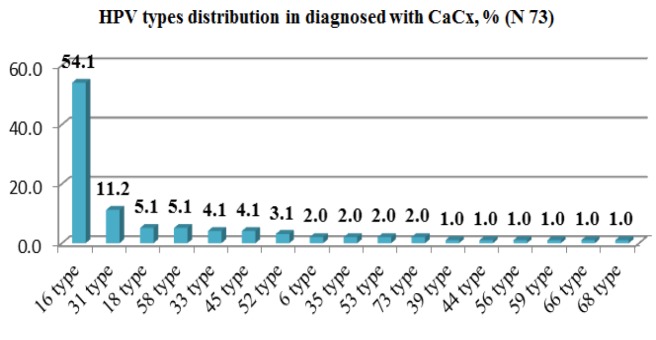
HPV Types Distribution in CaCx Diagnosed Women

**Figure 5:**
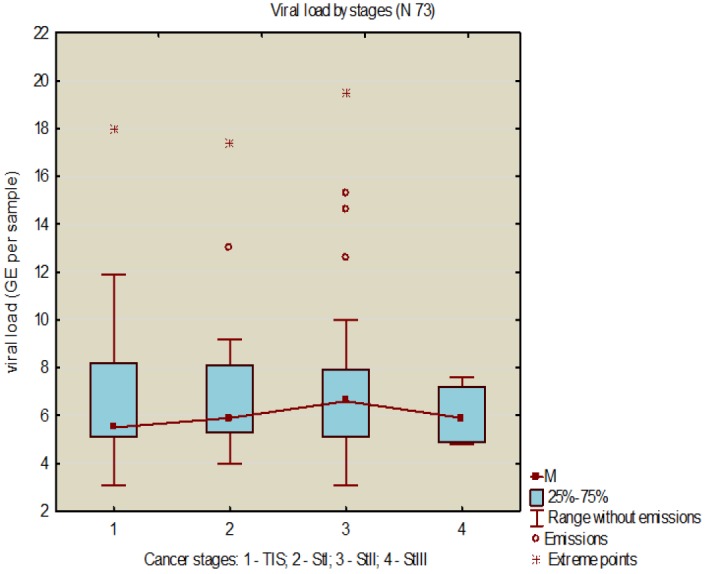
Relationship between the HPV Viral Load and the CaCx Stages

**Table 2:** Distribution of Viral Load by Age Groups

	Results							
Variable	Age groups	N observ.	Average	95% CI	95% CI	Minimum	Maximum	SD.
Viral load, 10^3^ GE/ per sample	1 – 18/29	123	5.65	5.01	6.29	1	20.8	3.53
	2 – 30/39	90	5.66	4.73	6.59	1	22.3	4.33
	3 – 40/49	42	5.61	4.45	6.75	1	19.8	3.54
	4 – 50/60 +	36	4.82	3.61	6.03	0.9	15.9	3.42

**Table 3:** HPV Prevalence, Leading Types and the Average Viral Load by Sites

Parameters	Маngystau	Аktobе	Аtyrau	Uralsk
N examined	249	411	242	264
Overall HPV prevalence, % (95% CI)	19.3	23.6	28	29.5
	(14.5;24.2)	(19.7;28.6)	(22.8;34.1)	(24.0;34.9)
	p=0.049	p=0.044	p=0.056	p=0.054
Average viral load (range)	5.9±3.4	6.4±4.6	5.4±3.8	4.5±2.7
	(2.0-18.7)	(0.9-21.1)	(1.0-22.3)	(1.0-16.4)
Leading HPV types by provinces, %	16 – 12.8%	16 – 26.3%	16 – 23.5%	16 – 29.6%
	18 – 12.8%	31 – 16.7%	31 – 16.2%	6 – 10.3%
	31 – 12.8%	51 – 10.3%	66 – 13.2%	52 – 10.3%
	51 – 10.6%	52 – 8.9%	45 – 10.3%	58 – 9.0%
	33 – 8.5%	18 – 8.0%	6 – 8.9%	39 – 7.7%
Average number of the types detected in one swab, %	1 – 72.0%	1 – 76.5%	1 – 85.3%	1 – 80.7%
	up to 4 – 28.0%	up to 6 – 23.5%	up to 5 – 14.7%	up to 3 – 19.3%

*Overall prevalence of HPV infection was determined *as 25.0% (22.3;27.7 95% CI, p = 0.05), or total 291 infected with HPV out of 1,166. Types distribution is summarized in Figure 1.

Top-5 leading HPV types were identified: 16 (26.4%); 31 (10.1%); 51 (9.4%); 52 (9.0%); 6 (7.9%). Of these, only type 6 refers to weakly carcinogenic, the rest are of the HR group. Type 6 matters as a causative factor for fast growing number of laryngeal cancers in Kazakhstan (Zatonskikh et al., 2016). 236 HPV positive women out of 291 in total (81.2%) were infected with types of the HR group (13 types out of a total 21). The highest prevalence of HPV was traced in the 18-29 years old group (42.9%), and decreased with age, that was a commonplace in all reports on the topic. Ratio of the HR group to non-HR by age categories increased from 1.6/1 in the youngest group up to 2.3/1 in the group of 40-49 years. Figure 2 shows the distribution of HPV types by age groups. 

Viral load for each HPV positive assay (total 291) was determined. Average viral load across the region appeared to be 5.5±3.8 (CI 95% 5.1;5.9) with a range of 0.9 to 22.3, Me 5.2 (3.1-8.4 by 25/75 quartile), and considered high for the general sample. Analysis of viral load by age groups revealed a weak inverse correlation (r = - 0.1, p< 0.005). However, relationship of this index and the age of HPV infected appeared not to be linear. Distribution of viral load by age categories is summarized in Table 2 and represented graphically in Figure 3. 

The graph demonstrates that the amount of abnormally high values is the greatest in the group of 30-39 years, when, according to body of numerous evidences, the viral persistence and the CIN process is initiated (Cuzick et al., 2008).

Whilst data collecting by provinces (oblasts), differences in the HPV prevalence were revealed, with a magnitude of 19.3% in Mangystau up to 29.5% in Uralsk. To determine the differences, a general sample is analyzed in the context of provinces. Data on the HPV infection by sites, including leading types and an average viral load are summarized in Table 3.

As follows from the Table 1, samples across the provinces are not homogeneous in respect to some basic parameters:

- 3.6% of the Caucasian ethnicity representatives in Mangystau vs. 0% in Uralsk;

- 19.3% of Slavic representatives in Uralsk vs. 11.6-12.0% in other areas.

In fact, differences in the ethnic composition of the study participants are inherent for these sites: proportion of the Slavic population in Uralsk or the Caucasians in Mangystau is traditionally higher due to the borderline nature of mentioned areas. Contingency analysis did not reveal associations whatever significant, but confirmed the sites heterogeneity (p = .002, Cramer’s V 0.23). Yet, a weak trend towards relationship between ethnicity and a specific type of HPV was established in the Mangystau (p = .083, Cramer’s V 0.13), confirming the data of the aforementioned pilot project of 2014, when quite a robust correlation had been found between Caucasian ethnicity and HPV 33 (p = .032, Cramer’s V 0.17). No correlations were established in other areas, though previous project revealed a relationship between Slavic ethnicity and type 45 in Uralsk (p = .0028, Cramer’s V 0.175). Associations between specific HPV types were found in multipositive assays: two times more frequent between 16 and 31 types (p < .001, Cramer’s V 0.18).

*CaCx sample*

In total, 73 women with the first time diagnosed cervical cancer were tested: 7 from Mangystau; 3 - from Atyrau; 8 - from Uralsk and 55 from the Aktobe province (oblast). Besides, their allocation by the cancer stages was also extremely uneven – 15 of Tis, 25 of StI (a/b), 27 of StII (a/b) and 6 of StIII respectively. This circumstance was related to difficulties in arranging the data collecting on sites. As such, contingency analysis by sites and CaCx stages was not performed due to uneven amount of samples. 

Average age of the tested was 49.0±12.4 (45.9;52.1, 95% CI), Me 47.5 (40.0 - 58.5 by 25/75 quartile; ranged 28 – 80). Distribution of HPV genotypes in patients with cervical cancer is shown in Figure 4.

One type of the virus was detected in 68.2%; 2 types – in 15.9%; 3 – in 9.1%; 4 – in 2.3% respectively. Ranking by all 98 identified types was as follows – 16 (54.1%), 31 (11.2%), 18 / 58 (5.1% each), 33 / 45 (4.1% each). In the context of types identified in each patient, the proportion of type 16 reached 72.6%, or otherwise, type 16 served as the cause for cancer development in more than 7/10 of all cases (53 infected out of 73 total). Besides, this type operated as single infection in 77.4% of cases. The most frequent associations were the following: type 16 / 31 (4 cases) and type 16 / 52 (3 cases). Type 16 appeared to be one of the most significant risk factor of the CaCx development (p=0.00007, phi 0.35, Pierson’s *X*^2^ 15.9).

Average viral load in the group of patients with cervical cancer was 6.9±4.0 (95% CI 6.1;7.7), and exceeded the average in the general sample (5.5±3.8, 95% CI 5.1;5.9). The range of the HPV viral load in CaCx patients appeared to be very variable (3.1-19.5). To clarify relationship between the cancer staging and the viral load, the stages by TNM / FIGO (The American Joint Committee on Cancer (AJCC) TNM / FIGO Classifications for Cervical Cancer, 2015) were converted into ordinal scale, with digital values ranging from 1 - Tis to 4 - St IIIab. A reliable relationship between the process staging and the viral load was found (p = 0.043, n = 73). The largest contribution to Pierson’s *X*^2^ was made by St IIa. The results are shown in Figure 5.

The viral load increased from the Tis to St IIab, demonstrating the largest extent in the Tis stage and the maximum number of emissions and extreme points in the St IIab stage. Given relatively small number of observations, rational explanation for such a distribution of the viral load has not been found, but it is quite reasonable to assume that the greatest extent in the Tis stage occurs due to the need to break a basal membrane, whereupon immune mechanisms are no longer able to restrain the replication of the virus. It is natural that as the process progresses towards St IIIab, the viral load decreases, since the macroorganism is no longer able to maintain adequate metabolism necessary for reproduction of the viral mass. Our limited observations on the behavior of the viral load depending on the degree of the oncological process have partially been confirmed by other researchers (Kim et al., 2008), who had showed that in persons with cervical cancer, the viral load was no longer to serve a predictor of the process progression like in CIN-II +, and did not depend directly on the process stage. To date, it has not been proven reliably that a lower viral load inevitably excludes the progressive disease (Dalstein et al., 2003). 

3 cases of cervical cancer detected at different stages and caused by types from the group 2b (possibly / probably carcinogenic) solely, are of the particular interest. A woman 60 years old from the Ural province with identified type 53, in the Tis stage and viral load of 6.0 GE*10^3^ / per sample; a 72-year-old woman from the Martuk district of the Aktobe province who was diagnosed with StIIb (T2bNxM0) cervical cancer, also caused by type 53 as a single infection, with viral load of 6.6 GE*10^3 ^/ per sample; a woman 80 years old from the Aktobe with identified type 73 and StIIb (T2bNxM0) stage of the process with viral load 7.3 GE*10^3^ / per sample. A high viral load, sole type of HPV of non-HR group, the estimated long exposure period given the age of these women were inherent to all of them. HPV types identification was confirmed by gene L1 sequencing in two cases - type 53 from Uralsk and type 73 from Aktobe. As to the third case, sequencing was failed. In fact, our report may be confirmed by the results of Korean researchers (Kim et al., 2014), who have found 2.4% HSIL with uncommon HPV genotypes (68, 26, 53, 66, 73, 44, 6, 11 and etc.) among 3,164 HPV-positive cases. We are prone to agree with the Chinese scientists reporting the CaCx mortality trends reaching a peak in the >85 yr age group (Du et al., 2015), and likely, the oldest age group will be the most vulnerable in future due to the cancers caused by 2b group (possibly / probably carcinogenic types).

## Discussion

Findings on undoubtedly high HPV prevalence among female population in the region (25.0%) with overwhelming majority of HR-HPVs were supported by other domestic researchers (Kairbayev et al., 2013), who found 25.1% HR-HPV positive results at N 2,408. The distribution diagram clearly indicates unconditional leading the most carcinogenic HPV type 16. Moreover, type 16 is the most prevailing in the group of having CaCx. Confirmed by other local scientists, domination of type 16 (Niyazmetova et al., 2016) calls for urgent need in revision of the current screening design. Accepted in the country cervical screening is featured by cytological methods only, whereas necessity to practice primary HPV screening has been evidenced worldwide, being to-date global trend (Basu et al., 2015; Nowakowsky et al., 2015; Italian MIDDIR Project on HPV test on primary screening, 2016; Smith et al., 2016). Potential to implement HPV primary screening scenario even in low-resource settings has been proved in a large-scale population-based the IARC’s project (Mittal et al., 2016). 

To our knowledge, vaccination is the only method aimed to prevent cervical cancer that proved its efficacy (Saslow et al., 2016). However, wide availability and acceptability of vaccination is still an unresolved issue for developing countries (Gupta et al., 2017). We believe that obtained results evidencing the role of type 16 as one of the most significant risk factors in CaCx development, convincingly substantiate the need to restore the adolescent girls vaccination program discontinued in the country in 2014. Even more so that a new nonavalent vaccine Gardasil-9 has come into a practice, being approved by FDA (FDA Approval Letter, December 10, 2014). It operates against 9 types of HPV (6, 11, 16, 18, 31, 33, 45, 52 and 58). First publications appeared in 2015, where the potential effect of the new vaccine was highly appreciated (Riethmuller et al., 2015). Along with, there are also categorically negative reviews that warn against the use of Gardasil-9 in teenage children (Fishman et al., 2014). 

On obtaining data on clear presence of malignant neoplasms caused by probably / possibly carcinogenic HPV types (type 53 - 2 cases and type 73 – 1 case), we have received a certain though insufficient evidence allowing to agree with Arbyn et al., that the need to reassess the carcinogenic potential of known types of HPV is coming up. The HR-HPV group will likely be increased in future from 13-14 known today up to 20.

Basing on the results on high HPV prevalence, unconditional domination of type 16 among the CaCx patients, we believe that the current situation calls for reassessing approaches to the CaCx prevention in the country. The present study allows for making a nationwide map on types distribution in the country, and thus contributes to the rational decision making in regard to transition towards HPV primary screening of cervical cancer. 

## Strengths and limitations of the study

Our aforementioned previous research of 2014 had a lot of limitations, and the first was its targeted group, as the HR-HPV suspected individuals only were tested. The present study, as being screening-like by its type, was designed to avoid such a bias, and its results quite met requirements relating to generalizability. 

Of limitations, the most serious is relatively low translatability of the study results due to restricted number of HPV types which are detectable by the Russian test systems (21 type) vs. HC-II or Inno Lipa systems (up to 44 types) mostly applying in similar researches. 

## Funding statement

This research was performed in frames of the scientific Project “Epidemiological analysis of Human Papillomavirus in Western Kazakhstan in relation to HPV-attributable cervical pathology - social, clinical and genetic aspects”, funded by the Committee of Science of the Ministry of Education and Science of the Republic of Kazakhstan (Grant № 2230/GF4, State registration No. 0115РК01224).

## Authors’ contributions

SB and AT were responsible for general editing of the manuscript and key issues of the research design. SB was major contributor to writing all sections of the manuscript. AK, SU and OU were responsible for editing the “Background” and “Methods” section. DZ and KS were responsible for selection of references and participated in writing the “Results” sections. 

All authors read and approved the final manuscript.
